# Particularities of the Theory of Mind in the Academic environment during the Covid 19 pandemic

**DOI:** 10.1192/j.eurpsy.2022.1519

**Published:** 2022-09-01

**Authors:** S. Ursoniu, C. Bredicean, C. Giurgi-Oncu, A.I. Bucur, I.-A. Rivis, C.L. Serban, I. Papava

**Affiliations:** 1 Victor Babes University of Medicine and Pharmacy, Functional Sciences, Timisoara, Romania; 2 Victor Babes University of Medicine and Pharmacy, Neuroscience, Timisoara, Romania; 3 Carol Davila University of Medicine and Pharmacy, Neuroscience, Bucharest, Romania

**Keywords:** medical students, covid 19, Emotions, Theory of Mind

## Abstract

**Introduction:**

The current period is marked by several negative aspects of the COVID 19 pandemic, which have led to a series of emotional and cognitive changes that affect our functioning. The ability to “read” the minds of others is the key aspect of social behavior, helping us understand our context.

**Objectives:**

To identify the level of emotion recognition in Medicine students during the Covid 19 pandemic.

**Methods:**

Throughout 2021, we evaluated 649 Romanian General Medicine students in years 4, 5 and 6, by using a Google Play application (android and iOS). We analyzed socio-demographic parameters and the affective component of Theory of Mind (The Reading the Mind in the Eyes Test). The mean scores between groups were compared with the Student’s t and the ANOVA tests.

**Results:**

The mean score was 25.83 ± 3.36 (min 11, max 33) out of a possible maximum of 36. We noted that women have a higher capacity for recognizing emotions than men (26.04 ± 3.22 vs. 25.01 ± 3.78, P = 0.0016) without differences in terms of the study year. Also, women showed a greater ability to recognize negative emotions compared to men (16.57 ± 2.44 vs. 15.49 ± 2.75, P <0.001). No statistically significant differences were found between the two genders in terms of positive emotions’ recognition.

**Conclusions:**

The ability to recognize emotions seems to be more developed in women, especially when it entails recognizing negative emotions. Theory of Mind abilities are important for empathy and the therapeutic relationship required in Medicine.

**Disclosure:**

No significant relationships.

